# Recognition of Mitochondrial Proteins in Plasmodium Based on the Tripeptide Composition

**DOI:** 10.3389/fcell.2020.578901

**Published:** 2020-09-16

**Authors:** Haodong Bian, Maozu Guo, Juan Wang

**Affiliations:** ^1^School of Computer Science, Inner Mongolia University, Hohhot, China; ^2^School of Electrical and Information Engineering, Beijing University of Civil Engineering and Architecture, Beijing, China; ^3^Beijing Key Laboratory of Intelligent Processing for Building Big Data, Beijing, China; ^4^Stage Key Laboratories of Reproductive Regulation & Breeding of Grassland Livestock, Hohhot, China

**Keywords:** malaria, Plasmodium, mitochondrion, tripeptide composition, support vector machine

## Abstract

Mitochondria play essential roles in eukaryotic cells, especially in Plasmodium cells. They have several unusual evolutionary and functional features that are incredibly vital for disease diagnosis and drug design. Thus, predicting mitochondrial proteins of Plasmodium has become a worthwhile work. However, existing computational methods can only predict mitochondrial proteins of *Plasmodium falciparum* (*P. falciparum* for short), and these methods have low accuracy. It is highly desirable to design a classifier with high accuracy for predicting mitochondrial proteins for all Plasmodium species, not only *P. falciparum*. We proposed a novel method, named as PM-OTC, for predicting mitochondrial proteins in Plasmodium. PM-OTC uses the Support Vector Machine (SVM) as the classifier and the selected tripeptide composition as the features. We adopted the 5-fold cross-validation method to train and test PM-OTC. Results demonstrate that PM-OTC achieves an accuracy of 94.91%, and performances of PM-OTC are superior to other methods.

## 1. Introduction

The parasite Plasmodium is the main cause of malaria, and kills more than one million African children annually (Phillips et al., 2017). There are approximately 40% humans whose are infected by malaria in the world. Four species of Plasmodium that can infect humans with malaria are *P. falciparum, Plasmodium vivax, Plasmodium malaria*, and *Plasmodium ovale*, respectively, where *P. falciparum* is the most lethal (Gardner et al., 2002). Research on the mitochondrial evolution and functions of Plasmodium indicates that Plasmodium mitochondrion is suitable targets for anti-parasitic drugs (Vaidya and Mather, 2009). Thus, it is exceptionally important to predict mitochondrial proteins of Plasmodium.

Traditional methods for predicting protein functions are based on biological experiments and they are costly and time-consuming. So, the researchers proposed the computational methods to predict the protein functions (Wei et al., 2015, 2017; Qu et al., 2019). The machine learning algorithms can achieve the prediction (Zou, 2019). There are two main steps of the machine learning: classifier and feature. For the classifiers, Logistic Regression (LR) has been used to recognize protein subcellular localization (Wan et al., 2015). Naïve Bayes (NB, Rish, 2001) is applied to predict protein-protein interaction sites (Murakami and Mizuguchi, 2010). As an efficient and powerful machine learning algorithm, SVM (Vapnik, 2000) has been applied widely for predicting protein subcellular localization (Hua and Sun, 2001; Kumar et al., 2018), G-protein coupled receptors (Karchin et al., 2002; Bhasin and Raghava, 2004a), protein-protein interactions prediction (Guo et al., 2008), and protein fold recognition (Ding and Dubchak, 2001).

For the features, there are many methods for extracting features from the protein primary sequences. The methods for extracting features of the amino acid, dipeptide and tripeptide from protein sequences can generate fixed-length data for the protein sequences with different length. Nakashima and Nishikawa (1994) first proposes the amino acid composition (AAC) with 20 dimensional vectors, i.e., the frequency of each single amino acid, to represent proteins. Dipeptide composition (DPC) with 400 dimensional vectors, i.e., the frequency of each one pair of amino acids in protein sequences, is used to discriminate protein subcellular localization (Bhasin and Raghava, 2004b; Ahmad et al., 2016). Furthermore, the researchers propose 8000 dimensional tripeptide composition (TPC) based on the structural properties of proteins, which has been used to predict protein subcellular localization (Liao et al., 2011) and sub-chloroplast localization (Lin et al., 2013). Besides, split amino acid composition (SAAC), which divides a whole sequence into three parts: N-terminal, C-terminal, and a region between the two, i.e., the remaining amino acids between N- and C-terminal, is introduced (Chou, 2005) and used for mitochondrial proteins prediction (Kumar et al., 2006, 2018). Additionally, Shen and Chou (2006) proposes pseudo amino acid composition (PseAAC) based on the physicochemical characteristics of proteins. The researchers used the PSI-BLAST software to compute the position-specific score matrices (PSSM) for extracting evolutionary information from protein sequences (Altschul et al., 1997). And PSSM also has been used for proteins subcellular localization prediction (Rashid et al., 2007) and protein-protein interaction site prediction (Zeng et al., 2019).

There are lots of approaches for predicting mitochondrial proteins of *P. falciparum*. Bender et al. (2003) uses the neural network and the relative amino acid frequency to predict mitochondrial transit peptides (mTPs) of *P. falciparum*. Verma et al. (2010) applies the SVM to predict mitochondrial proteins of *P. falciparum* and uses SAAC and PSSM to represent protein sequences. Jia et al. (2011) uses Bi-profile Bayes and SAAC to extract protein sequence features and uses the SVM classifier as the classifier to train two models (PfMP-N25 and PfMP-30) in order to recognize mitochondrial proteins of *P. falciparum*. Chen et al. (2012) proposes an increment of diversity (ID) method based on the n-peptide composition of the reduced amino acid alphabet (RAAA) to predict mitochondrial proteins of *P. falciparum*. Furthermore, Ding and Li (2015) uses the analysis of variance (ANOVA) to reduce the feature dimension and generate the optimal 5-gap dipeptide composition from the protein sequences. However, all these methods mentioned above are used to predict only mitochondrial proteins of *P. falciparum*. Our paper will introduce a noval model, named by PM-OTC, to predict mitochondrial proteins of all Plasmodium. PM-OTC uses the SVM to classify and selects several tripeptides to represent the proteins. To evaluate the performance of PM-OTC, we adopted 5-fold cross-validation to train and test our method on the two datasets: the PM275 (collected from UniprotKB/SwissProt) and the PfM175 (used by Bender et al., 2003).

## 2. Materials and Methods

### 2.1. Datasets

#### 2.1.1. PM275

The proteins of PM275 are selected from UniprotKB/SwissProt (released 2020_01) by the following rules: (1) without ambiguous amino acids, such as “B,” “X,” and “Z;” (2) their function that have been confirmed by biological experiments; (3) sequences with > 50 length. Here we obtain 54 mitochondrial proteins as positive examples, and 340 non-mitochondrial proteins as negative examples, including cytosol proteins, secreted proteins, and apicoplast proteins. Next we used the CD-HIT (Fu et al., 2012) software with global alignment and sequence identity threshold set to 0.4 to process negative sequences in order to eliminate the similar sequences. Then the protein sequences whose sequence similarity is more than or equal to 40% are regarded as the same cluster and the longest sequence from each cluster are chosen the resulting sequences. Finally, we obtained 221 non-mitochondrial proteins as the negative examples. So, the PM275 contains 54 mitochondrial proteins and 221 non-mitochondrial proteins.

#### 2.1.2. PfM175

The PfM175 is mainly used in predict the mitochondrial proteins of *P. falciparum*. This dataset includes 40 mitochondrial proteins and 135 non-mitochondrial proteins (61 cytoplasmic, 21 secretory, and 53 apicoplast, respectively).

### 2.2. Sequences Representation

A protein sequence needs an efficient mathematical representation that can correctly express the inherent connection with the prediction types. To efficiently identify mitochondrial proteins of Plasmodium and build a robust model, we synthetically considered three sequences of features based on the protein primary sequence.

#### 2.2.1. AAC

AAC has low complexity and has been widely used to predict the function of proteins. Given a protein sequence *S* with *L* residue, AAC represents it as following:

(1) $$\begin{array}{l} AAC\left(S\right)={\left(f_{1},f_{2},\cdots \;,f_{20}\right)}^{T} \end{array}$$

where *f*_*i*_ = *n*_*i*_/*L* (*i* = 1, 2, ⋯ , 20), *n*_*i*_ is the frequency of the *i*-*th* amino acid.

#### 2.2.2. DPC

DPC computes the frequency of two amino acids. A protein sequence can be represented by a 400 dimensional vector. DPC contains information about the proportion of amino acids as well as the order of sequence.

(2) $$\begin{array}{l} DPC\left(S\right)={\left(f_{1},f_{2},\cdots \;,f_{400}\right)}^{T} \end{array}$$

(3) $$\begin{array}{l} f_{i}=\frac{dep\left(i\right)}{\sum dep\left(i\right)} \end{array}$$

where *dep*(*i*) is one out of 400 dipeptides, ∑*dep*(*i*) represents total number of all possible dipeptides in sequence *S*.

#### 2.2.3. TPC

TPC computes the frequency of three contiguous amino acids. A protein sequence can be represented by a 8000 dimensional vector.

(4) $$\begin{array}{l} TPC\left(S\right)={\left(f_{1},f_{2},\cdots \;,f_{8000}\right)}^{T} \end{array}$$

(5) $$\begin{array}{l} f_{i}=\frac{tep\left(i\right)}{\sum tep\left(i\right)} \end{array}$$

where *tep*(*i*) is one out of 8000 tripeptides, ∑*tep*(*i*) represents total number of all possible tripeptides in sequence *S*.

### 2.3. Support Vector Machine

SVM is a powerful and efficient machine learning algorithm for linear, non-linear classification and regression. Compared with other machine learning algorithms, the advantage of the SVM algorithm is that the dimension of SVM parameters equals the number of training samples (Zavaljevski et al., 2002).

SVM algorithm aims to calculate an optimal hyperplane that can separate two samples correctly in space. The optimal hyperplane, also known as support vector, is a set of vectors obtained by maximizing the separating margin on the training set. For linear separable classification problems, the optimal hyperplane can be directly obtained by the constrained optimization problem. For non-linear classification, the advantage of SVM is to introduce kernel function and transform the non-linear classification problem into a linear classification problem (Amari and Wu, 1999; Hofmann et al., 2008). The essence of kernel function or kernel technique is to map Euclidean space to Hilbert space by non-linear transformation so that the non-linear classification problem of original space can be transformed into the linear classification problem of calculating the optimal hyperplane in high dimensional space. Scikit-learn (Pedregosa et al., 2011), which provides linear, Gaussian (RBF), polynomial, and sigmoid kernel function, are adopted to implement the SVM classifier. We mainly use linear kernel function and Gaussian kernel function in our experiments. In order to find the optimal values of the two parameters C and γ, we employ a grid search method with 5-fold cross-validation. The range of C and γ are [2^−1^, 2^3^] and [2^−4^, 2^−1^] with the step of 2.

### 2.4. Feature Selection

TPC can obtain 8000 feature values for a protein. However, these feature values may contain redundant and noisy information which will affect the training model and can lead to low prediction accuracy eventually. Accordingly, it is vital to select appropriate features from TPC to improve the prediction accuracy. The analysis of variance (ANOVA) can filter out the tripeptides with low variance, which is suitable for processing TPC because of its lots of zero values. ANOVA can compute the difference in the mean of two or more samples. ANOVA computes a F-value by the difference within the same group and the difference among different groups (Anderson, 2001). The F-value for the ξ-*th* tripeptide is defined as:

(6) $$\begin{array}{l} F\left(\xi \right)=\frac{s_{B}^{2}\left(\xi \right)}{s_{W}^{2}\left(\xi \right)} \end{array}$$

where Sb2 and Sw2 are calculated by the following formulas:

(7) $$\begin{array}{l} s_{B}^{2}\left(\xi \right)=\overset{K}{\underset{i=1}{\sum }}m_{i}{\left(\frac{\sum_{j=1}^{m_{i}}f_{\xi }\left(i,j\right)}{m_{i}}-\frac{\sum_{i=1}^{K}\sum_{j=1}^{m_{i}}f_{\xi }\left(i,j\right)}{\sum_{i=1}^{K}m_{i}}\right)}^{2}/\left(K-1\right) \end{array}$$

(8) $$\begin{array}{l} s_{W}^{2}\left(\xi \right)=\overset{K}{\underset{i=1}{\sum }}\overset{m_{i}}{\underset{j=1}{\sum }}{\left(f_{\xi }\left(i,j\right)-\frac{\sum_{i=1}^{K}\sum_{j=1}^{m_{i}}f_{\xi }\left(i,j\right)}{\sum_{i=1}^{K}m_{i}}\right)}^{2}/\left(M-K\right) \end{array}$$

where K and M represent the number of groups and total number of samples. *f*_ξ_(*i, j*) is the frequency of the ξ-*th* tripeptide of the *j*-*th* sample in the *i*-*th* group. *m*_*i*_ represents the number of samples in the *i*-*th* group. The *F*(ξ) in Equation (6) computes the ratio of the sample variance among groups and the sample variance within groups. MSB (mean square between) denotes the sample variance between groups and MSW (mean square within) denotes the sample variance within groups. If the value of *F*(ξ) is away from 1, then there is a significant difference between MSB and MSW. On the contrary, if the value of *F*(ξ) is close to 1, then there is no significant difference between MSB and MSW. The larger the *F*(ξ) value is, the greater the impact of the ξ-*th* tripeptide on the predicted results. So we rank 8000 tripeptides in the descending order of *F*(ξ) values and employ an Incremental Feature Selection (IFS) strategy to find the optimized TPC with the highest prediction accuracy as the features. The detailed steps are as follows. First, we choose the tripeptide with the highest F-value to generate an initial feature set. Second, we select another tripeptide with the second-highest F-value and add it to the initial feature set and form a new feature set. And repeat this step to form 8000 feature sets. Each feature set is used to train and test a prediction model. Finally, we choose the feature set, which prediction model based on it achieve the maximum accuracy, as the optimized TPC.

### 2.5. Performance Measures

We use 5-fold cross-validation to assess the prediction performance of our method. First, we randomly divide the dataset into five mutually exclusive subsets of similar size. Second, we choose one subset as the testing dataset and the other four subsets as the training dataset. So, we run five times of training and testing, and return the average value of five test results.

Here, six metrics for evaluating methods are used, accuracy, sensitivity, precision, recall, F-score, and the Matthews correlation coefficient (MCC), respectively. The detailed formulas are followings:

(9) $$\begin{array}{l} Accuracy=\frac{\text{TP}+\text{TN}}{\text{TP}+\text{TN}+\text{FP}+\text{FN}} \end{array}$$

(10) $$\begin{array}{l} Sensitivity=\frac{\text{TP}}{\text{TP}+\text{FN}} \end{array}$$

(11) $$\begin{array}{l} precision=\frac{\text{TP}}{\text{TP}+\text{FP}} \end{array}$$

(12) $$\begin{array}{l} recall=\frac{\text{TN}}{\text{TN}+\text{FP}} \end{array}$$

(13) $$\begin{array}{l} F-score=\frac{2*precision*recall}{precision+recall} \end{array}$$

(14) $$\begin{array}{l} MCC=\frac{\text{TP}\times \text{TN}-\text{FP}\times \text{FN}}{\sqrt{\left(\text{TP}+\text{FP}\right)\left(\text{TP}+\text{FN}\right)\left(\text{TN}+\text{FP}\right)\left(\text{TN}+\text{FN}\right)}} \end{array}$$

Here TP represents the number of mitochondrial proteins predicted correctly, FP represents the number of non-mitochondrial proteins predicted incorrectly, TN represents the number of non-mitochondrial proteins predicted correctly, FN denotes the number of mitochondrial proteins predicted incorrectly.

## 3. Results

Experiments first evaluate the performances of using AAC, DPC, TPC as the features, and the different machine learning algorithms as classifiers. Results demonstrate that TPC performs better than other feature sets (Tables 1, 2). So, we use TPC as features and then use the ANOVA to select a part of TPC as features in order to improve the prediction accuracy. Experiments then evaluate the performances of using the optimized TPC as the features. Results suggest that the optimized TPC significantly improves the accuracy of discriminating mitochondrial proteins of Plasmodium, especially for the SVM classifier (Table 3). Therefore, we obtain a new mode named PM-OTC, using the SVM as the classifier and the optimized TPC as the features, to predict the mitochondrial proteins of Plasmodium. Experiments finally evaluate the performance of PM-OTC by comparing PM-OTC with other computational methods (PlasMit, PFMpred, PfMP-N25, PfMP-30, ID, and Ding). Results show PM-OTC is superior to the others (Table 4).

**Table 1:** Cross-validation performances of AAC with different classifiers on PM275.

**Classifier**	**Accuracy**	**Sensitivity**	**Precision**	**Recall**	**F-score**	**MCC**
LR	**82.91%**	12.73%	**71.26%**	56.36%	**55.28%**	**0.24**
NB	57.82%	**58.73%**	58.16%	**58.02%**	50.68%	0.12
SVM	80.36%	1.82%	40.18%	50%	44.56%	0

*The bold values are the maximum value for each column*.

**Table 2:** Cross-validation performances of DPC, TPC, and combination features using different classifiers on PM275.

**Feature vector**	**Classifier**	**Accuracy**	**Sensitivity**	**Precision**	**Recall**	**F-score**	**MCC**
	LR	83.27%	33.27%	76.59%	64.36%	66.84%	0.39
DPC	NB	60.73%	**86.73%**	63.55%	70.49%	57.46%	0.33
	SVM	86.18%	29.45%	92.68%	72.73%	68.33%	0.49
	LR	82.55%	31.45%	74.25%	63.23%	65.32%	0.35
AAC+DPC	NB	60.73%	**86.73%**	63.61%	70.48%	57.42%	0.33
	SVM	85.82%	27.64%	92.52%	63.82%	67.18%	0.48
	LR	88.73%	42.55%	93.91%	71.27%	75.54%	0.60
TPC	NB	82.91%	12.73%	81.24%	56.36%	56.09%	0.29
	SVM	**89.82%**	48.18%	**94.43%**	**74.09%**	**78.80%**	**0.65**

*The bold values are the maximum value for each column*.

**Table 3:** Cross-validation performances of optimized TPC using different classifiers on PM275.

**Classifier**	**Feature** **dimension**	**Accuracy**	**Sensitivity**	**Precision**	**Recall**	**F-score**	**MCC**
LR	984	91.27%	55.64%	95.13%	77.82%	82.8%	0.71
NB	2578	85.09%	24%	92.22%	62%	63.98%	0.43
SVM	399	**94.91%**	**74.18%**	**97.05%**	**87.09%**	**90.86%**	**0.83**

*The bold values are the maximum value for each column*.

**Table 4:** Cross-validation performance of PM-OTC compared with other methods on PfM175.

**Method**	**Accuracy**	**Sensitivity**	**Recall**	**MCC**
PlasMit (Bender et al., 2003)	90.00%	94.00%	89.00%	0.74
PFMpred (Verma et al., 2010)	92.00%	97.50%	90.40%	0.81
PfMP-N25 (Jia et al., 2011)	96.00%	87.50%	98.50	0.93
PfMP-30 (Jia et al., 2011)	98.80%	97.50%	**99.30%**	0.97
ID (Chen et al., 2012)	92.00%	**100%**	89.63%	0.82
Ding (Ding and Li, 2015)	97.10%	90.00%	**99.30%**	0.92
Our method	**99.43%**	97.50%	98.75%	**0.98**

*The bold values are the maximum value for each column*.

### 3.1. Analysis of AAC on PM275

**Figure 1:**
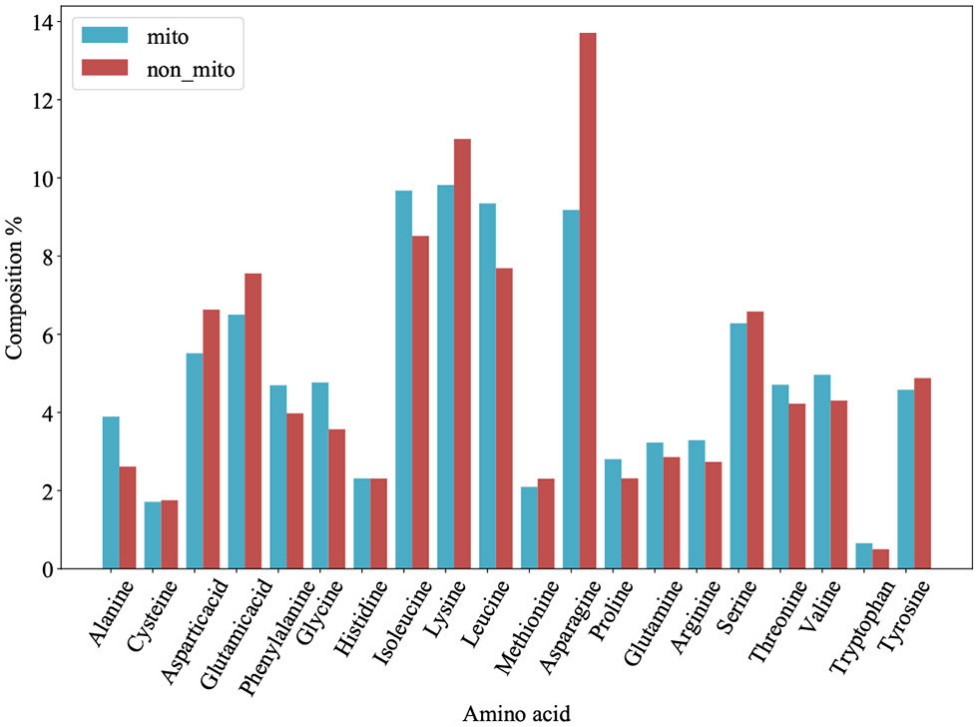
Amino acid composition of 54 mitochondrial proteins (mito) and 221 non-mitochondrial proteins (non_mito). The abscissa represents the abbreviation of amino acid, and the ordinate represents the percentage content of amino acid.

We plot a histogram based on the frequency of each amino acid for each protein from PM275 (Figure 1) in order to analyze the differences between mitochondrial proteins and non-mitochondrial proteins. Figure 1 shows that mitochondrial proteins have more alanine, phenylalanine, glycine, isoleucine, leucine, proline, glutamine, arginine, threonine, and valine than non-mitochondrial proteins. On the contrary, non-mitochondrial proteins have more aspartic, glutamic, lysine, asparagine, serine, and tyrosine than mitochondrial proteins. Only the amino acid cysteine and histidine are no significant differences in mitochondrial and non-mitochondrial proteins. We further research the prediction performance when using the AAC as the features. So, we extract the AAC (Equation 1) for each protein from PM275. Here we use the SVM, the Logistic Regression (short for LR) and the Naïve Bayes (short for NB) as classifiers.

Table 1 shows the results. The results show that the LR has a more excellent performance than other classifies in accuracy of 82.91%, precision of 71.26%, F-score of 55.28%, and MCC of 0.24. The NB performs better in sensitivity (58.73%) and recall (58.02%) than other classifiers. Overall, using the AAC as the features to predict mitochondrial proteins of Plasmodium have low performance.

### 3.2. Prediction Performances of DPC, TPC and Combined Feature on PM275

Next, we consider three feature sets: DPC (Equations 2, 3), DPC combined with AAC, and TPC (Equations 4, 5). We input these three feature sets into three classifiers (LR, NB, SVM). Results are recorded in Table 2. Table 2 shows that the model using the SVM as the classifier and the TPC as the features performs better in almost all measures than other models, and achieves the accuracy of 89.82%, precision of 94.43%, recall of 74.09%, F-score of 78.80%, and MCC of 0.65. The models using TPC as the features get better performances compared with DPC and AAC+DPC. Thus, TPC has obvious advantages in discriminating mitochondrial proteins of Plasmodium. Table 1 shows that the SVM classifier does not perform as well as the other two classifiers when using the AAC as the features. However, as we can see from Table 2, the SVM classifier performs efficient and powerful when using high dimensional feature sets as the input features. So, we choose TPC as the features.

### 3.3. Prediction Performance of Optimized TPC on PM275

**Figure 2:**
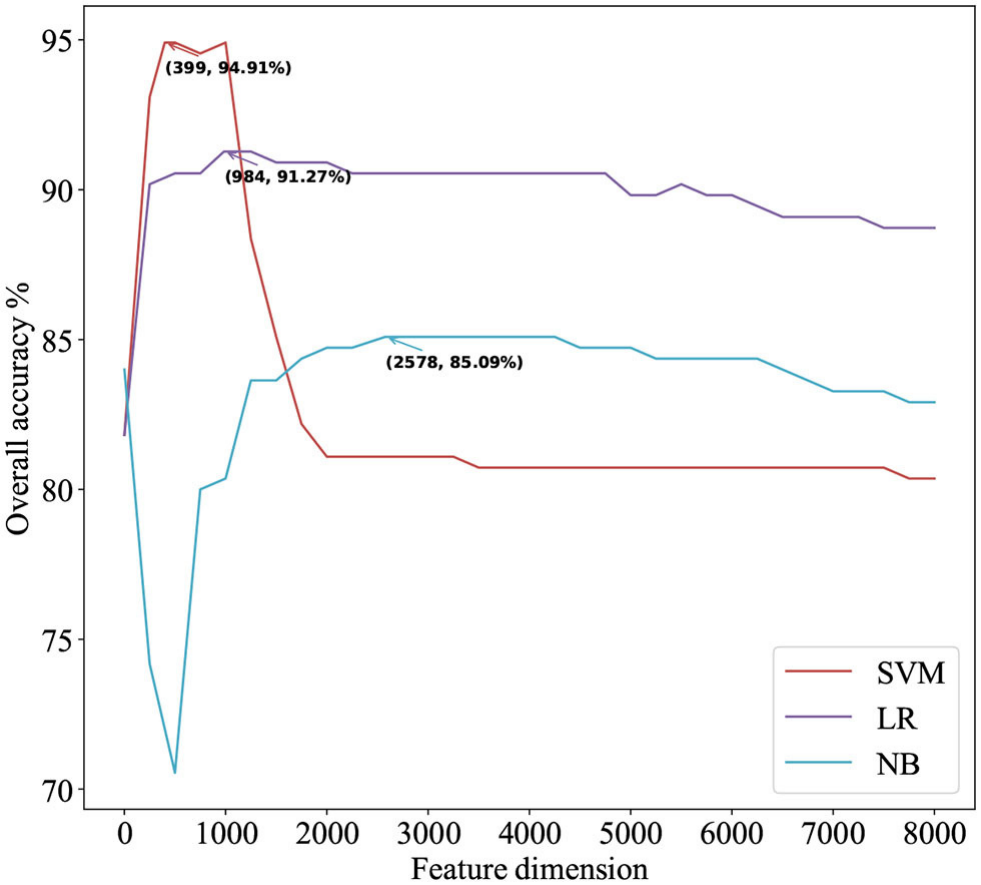
The IFS curve for predicting mitochondrial proteins of Plasmodium using three classifiers. The accuracies of the SVM classifier and the LR classifier improve when the number of features is initially increased. When the number of features exceeds 399, the accuracy of the SVM classifier decreases significantly and finally returns to stable. With the increase in the number of features, the accuracy of the NB classifier first decreases significantly and then gradually increases to stable.

**Figure 3:**
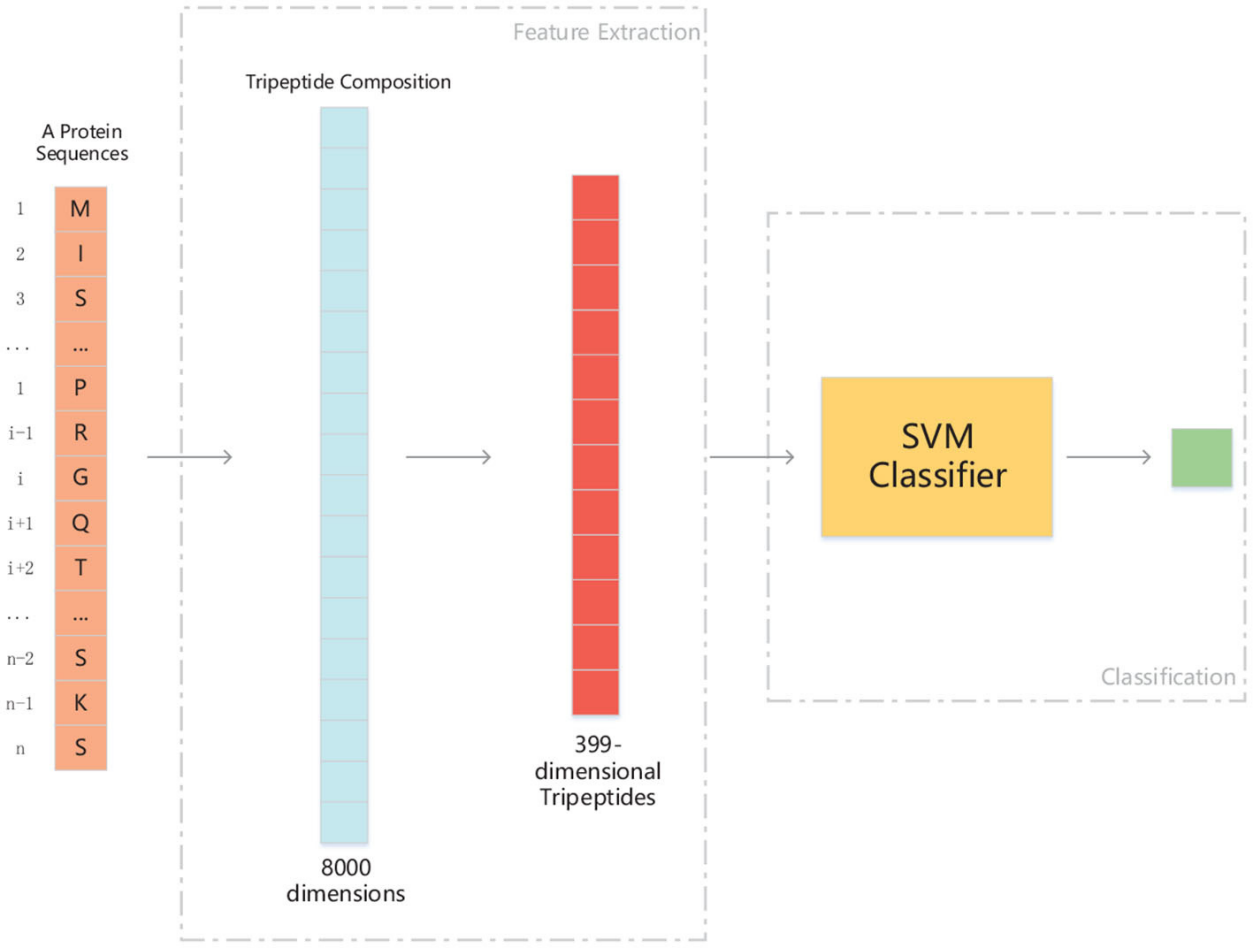
The structure of PM-OTC model. The input data is whole protein sequence. First, through extracting tripeptides from raw sequence, 8000-dimensional TPC are obtained. And then, TPC constitute a feature vector of 399 dimensions by ANOVA, which is fed into the SVM classifier for prediction.

We use ANOVA and IFS strategy to reduce the dimension of TPC and further obtain the optimized TPC as the features. We rank the 8000 dimensional TPC according their F-value (Equation 6) and adopt IFS to generate 8000 subsets. Then we input all 8000 subsets into three classifiers (LR, NB, SVM) and calculate the accuracy of 5-fold cross-validation of each subset. Figure 2 shows the IFS curve. From Figure 2, we can see that the accuracy of 5-fold cross-validation has the maximum 94.91% when using the SVM as the classifier. And the optimized TPC only contains 399 tripeptides. Table 3 shows that the model using the SVM as the classifier and the optimized TPC as the features performs more reliable than the other classifiers. Accordingly, the PM-OTC model uses the top 399 ranked tripeptides as features and the SVM as the classifier to predict the mitochondrial proteins of Plasmodium. Figure 3 shows the structure of PM-OTC.

Table 2 shows that the model using the SVM as the classifier and the TPC as features can only achieve the cross-validated accuracy by 89.82%, which is lower than that (94.91%) of PM-OTC. So, the optimized TPC significantly enhances the prediction performance of the model using SVM as the classifier, especially in accuracy, and improves it by 5.09%. This result shows that the original 8000 dimensional TPC includes redundant or noise information. Table 2 shows that the modes using the LR and NB as the classifiers and the optimized TPC as the features also improve the prediction performance (Figure 3). This result shows that the optimized TPC is an effective and efficient feature vector in predicting mitochondrial proteins of Plasmodium.

### 3.4. Performance of the PM-OTC on PfM175

Most of the published methods can only predict mitochondrial proteins of *P. falciparum* and built on PfM175. Accordingly, we train and test PM-OTC adopting 5-fold cross-validation on PfM175 in order to compare our approach with other computational methods. Table 4 records the comparison of all methods. Result shows that our method outperforms other methods with an accuracy of 99.43% and MCC of 0.98. Meanwhile, precision and F-score of our method are 99.64 and 99.15%, respectively. This result indicates that the prediction results of PM-OTC are more correct and reliable than other approaches.

## 4. Conclusion

Predicting mitochondrial proteins of Plasmodium is the key to treating malaria because mitochondrion is a suitable target for anti-malarial drugs. Here we build the PM-OTC to predict the mitochondrial proteins of Plasmodium instead of only predicting mitochondrial proteins of *P. falciparum*.

The PM-OTC uses the optimized TPC as the features and the SVM as the classifier to predict mitochondrial proteins of Plasmodium. The performance of PM-OTC on PM275 indicates that PM-OTC performs well in predicting mitochondrial proteins of Plasmodium with an accuracy of 94.91%. The performance of PM-OTC on PfM175 shows that PM-OTC improves the accuracy by 0.64−9.43% compared with other methods. So, the PM-OTC is efficient and effective in predicting mitochondrial proteins of *P. falciparum*.

## Data Availability Statement

All datasets presented in this study are included in the article/supplementary material.

## Software Available

The software of PM-OTC can download from https://github.com/CS-BhD/PMOTC.

## Author Contributions

HB proposed the method. HB and JW designed the experiments. All author wrote the manuscript.

## Conflict of Interest

The authors declare that the research was conducted in the absence of any commercial or financial relationships that could be construed as a potential conflict of interest.
